# A Case of Breast Cancer Arising From a Florid Adenosis Nodule

**DOI:** 10.7759/cureus.85725

**Published:** 2025-06-10

**Authors:** Kazusane Takaoka, Shoji Oura

**Affiliations:** 1 Department of Surgery, Kishiwada Tokushukai Hospital, Kishiwada, JPN

**Keywords:** breast cancer, florid adenosis, metachronous development, sclerosing adenosis, triple negative breast cancer

## Abstract

A 42-year-old woman noticed a left breast mass and was diagnosed with florid adenosis by vacuum-assisted biopsy (VAB) 10 years before. Semiannual ultrasound follow-ups showed gradual shrinkage of the florid adenosis nodule as time passed after VAB, but showed a slight increase in size after her marriage at the age of 40. Thereafter, the sudden rapid growth of the nodule made us again examine it in detail. Magnetic resonance imaging of the masses, including a daughter nodule in the nipple direction, showed low signals on T1-weighted images, slightly high signals on fat-suppressed T2-weighted images, and persistent rim enhancement on subtraction images. The patient underwent core needle biopsy under the tentative diagnosis of breast cancer. Pathological study showed atypical cells growing in a papillary fashion with bleeding and necrosis, leading to the diagnosis of invasive ductal carcinoma. Immunostaining showed estrogen and progesterone receptor negativity, human epidermal growth factor receptor type 2 negativity, and a high Ki-67 labeling index of 60%. The patient, therefore, underwent nipple-preserving mastectomy and sentinel biopsy followed by immediate breast reconstruction using an extended latissimus dorsi musculocutaneous flap. Postoperative pathological study showed that the breast cancer had similar pathological findings to those of the core needle biopsy specimen and a higher Ki-67 labeling index of 70%. The patient recovered uneventfully and was discharged on the 9th day after the operation. The patient has received dose-dense chemotherapy and is scheduled for periodical checkups on an outpatient basis. Breast specialists should note that even pathologically proven florid adenosis nodules might develop breast cancer.

## Introduction

Fibrocystic disease is histologically characterized by the coexistence of proliferative and regressive changes in epithelial and stromal components of the breast [1]. The hormonal environment of the patients plays a major role in the development of fibrocystic disease, causing various pathological conditions such as ductal/lobular hyperplasia, cyst formation, and fibrosis. Fibrocystic disease of the breast was once considered a risk factor for breast cancer, but is now considered “Aberrations of Normal Development and Involution” [2]. It, therefore, does not need any surgical intervention to properly manage fibrocystic disease.

Adenosis, one phenotype of fibrocystic disease, is a condition in which ductal proliferation occurs prominently in a certain area of the mammary gland and forms a lesion with relatively clear margins. Breast adenosis has three major subtypes in descending order of fibrous component ratios: sclerosing adenosis, blunt duct adenosis, and florid adenosis [3]. Sclerosing adenosis not only resembles scirrhous-type invasive ductal carcinoma on images but is also associated with a slight increase in breast cancer development [4,5], while no studies have reported the correlation between blunt duct adenosis / florid adenosis and breast cancer.

We herein report a very rare breast cancer case that metachronously developed in a pathologically confirmed florid adenosis nodule.

## Case presentation

**Figure 1:**
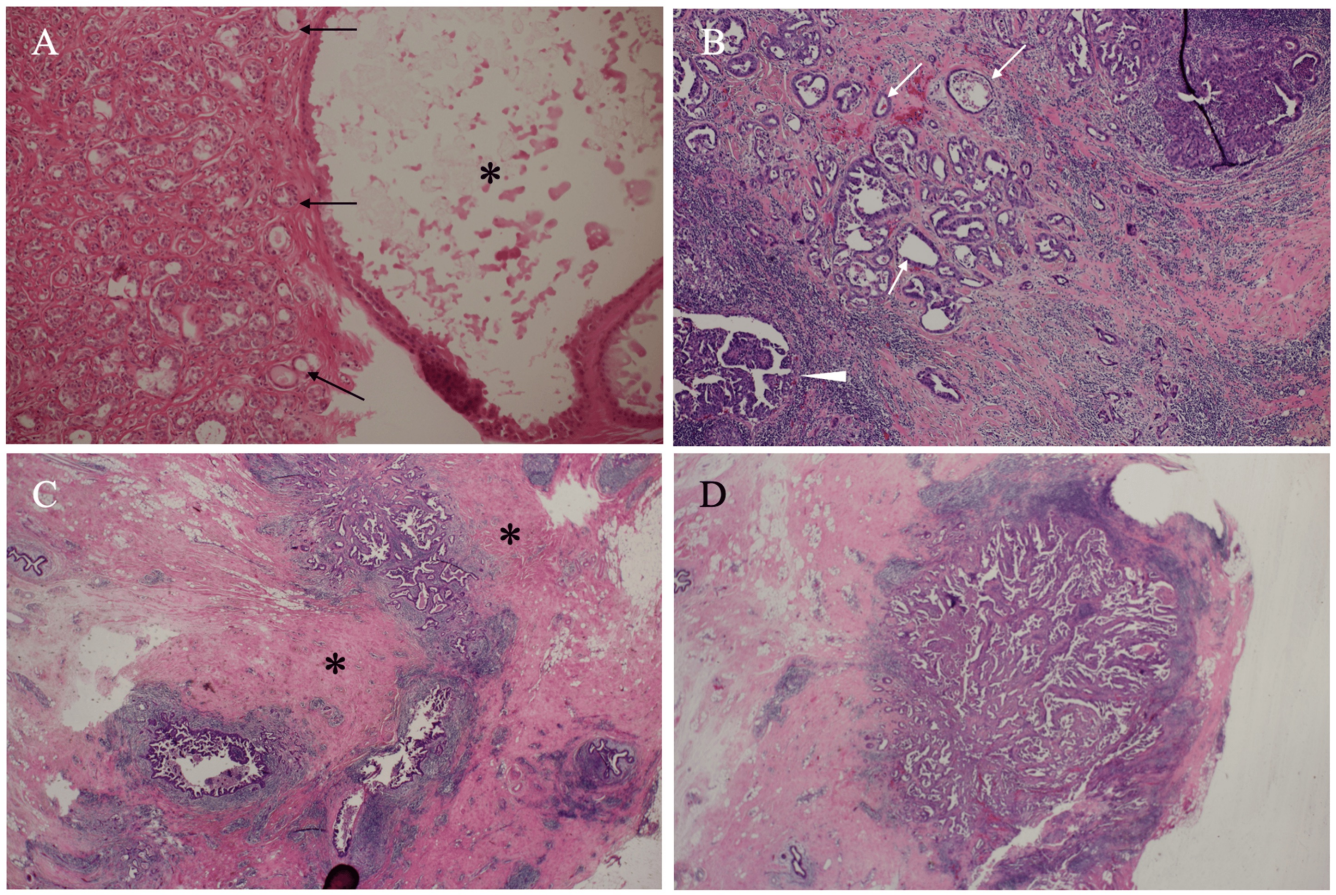
Pathological findings A) Magnified view of vacuum-assisted biopsy specimen pathologically showed a large cyst with apocrine metaplasia (asterisk) and marked ductal hyperplasia (arrows). B) Postoperative pathological study showed atypical cells growing in papillary (arrowhead) and tubular (arrows) fashions. C) Low magnified view pathologically showed abundant fibrous components (asterisks) around cancer cell clusters. D) The daughter nodule exhibited similar pathological features to those of the main tumor.

A 42-year-old nulliparous woman received a vacuum-assisted biopsy (VAB) for her left breast mass at the age of 32. Pathological study of the mass showed marked ductal hyperplasia and cyst formation with apocrine metaplasia, leading to the diagnosis of florid adenosis (Figure 1A).

**Figure 2:**
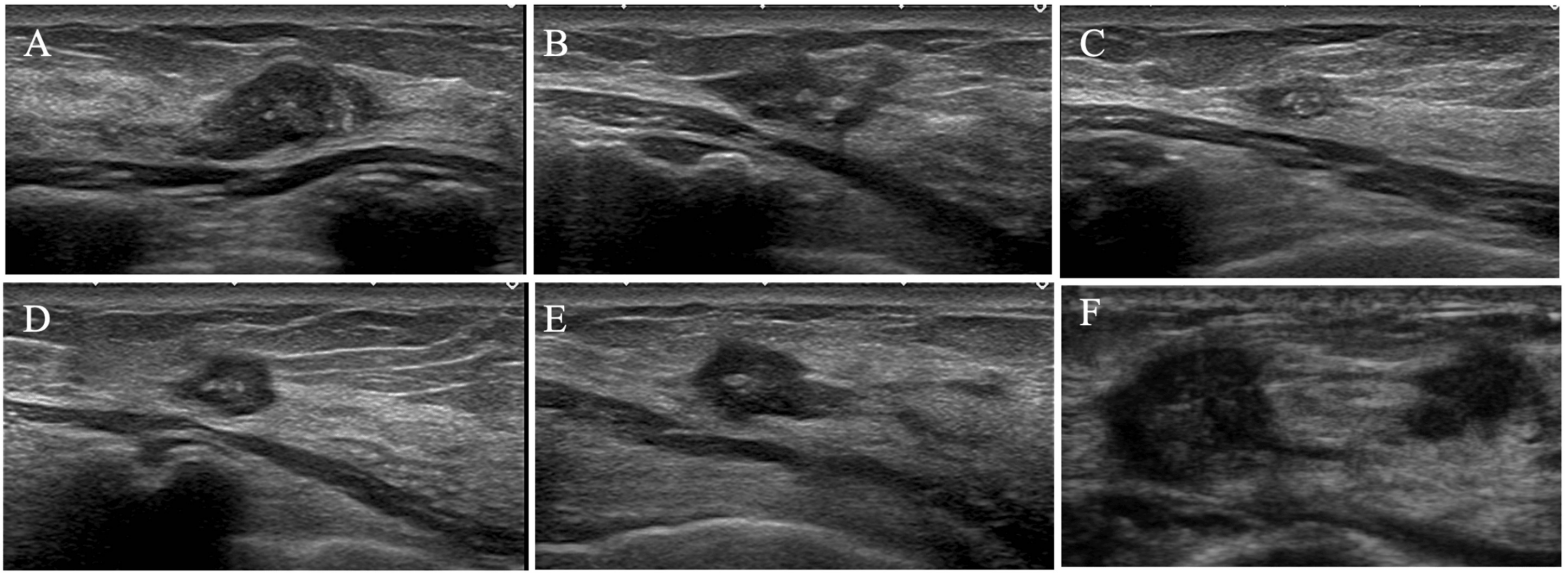
Ultrasound findings Ultrasound of the florid adenosis nodule showed continued shrinkage (A: 60 months, B: 72 months, and C: 78 months) after the vacuum-assisted biopsy, enlargement of the nodule from the 90th month (D) onwards (E: 114 months), and invasive carcinoma findings at 120 months (F).

Due to the lack of complete removal of the florid adenosis nodule, the patient requested us to semi-annually follow the mass on ultrasound. The nodule gradually shrank over time after VAB, but after her marriage at the age of 40, it showed a slight increase in size but suggested no definitive malignant ultrasound findings (Figure 2).

**Figure 3:**
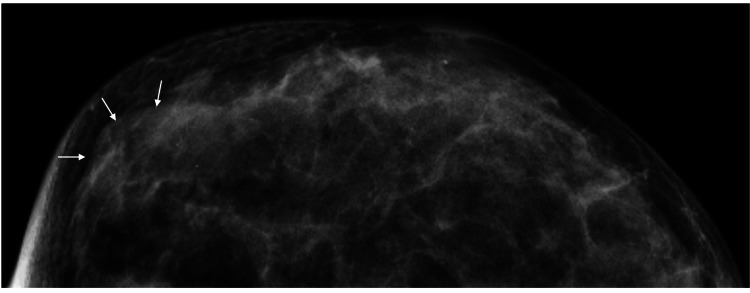
Mammography findings Mammography only showed focal asymmetric density (arrows) and did not depict any mass shadows.

The patient did not wish to have a baby and was not undergoing any hormonal treatment. Thereafter, the sudden rapid regrowth of the nodule made us again examine it in detail at her age of 42. Mammography showed no remarkable findings due to the dense breast (Figure 3).

**Figure 4:**
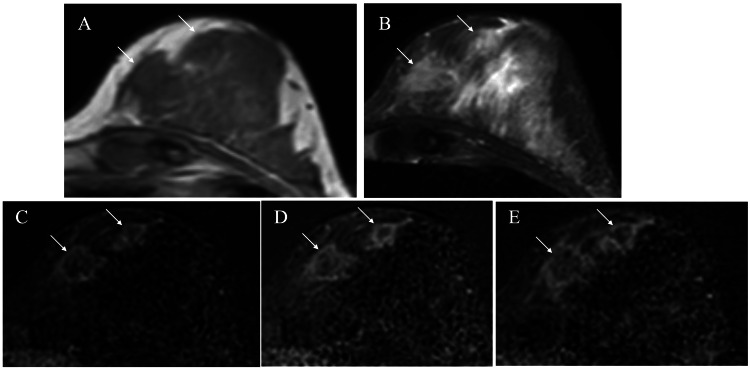
Magnetic resonance imaging (MRI) findings MRI of the masses (arrows) showed low signals on T1-weighted images (A), slight high signals on fat-suppressed T2-weighted images (B), and retained rim enhancement (C: early, D: mid, and E: late phases) on subtraction images.

Ultrasound showed an irregular mass, 15 mm in size, with indistinct margins, punctate echogenic foci against the low internal echoes, ruptured anterior borders of the mammary gland, and a daughter nodule in the nipple direction (Figure 2F). Magnetic resonance imaging (MRI) of the mass and the daughter nodule showed low signals on T1-weighted images, slightly high signals on fat-suppressed T2-weighted images, and persistent rim enhancement on subtraction images (Figure 4).

The patient, therefore, underwent core needle biopsy of the regrown florid adenosis nodule under the tentative diagnosis of breast cancer. Pathological study showed atypical cells growing in a papillary fashion with bleeding and necrosis, leading to the diagnosis of invasive ductal carcinoma. Immunostaining showed estrogen and progesterone receptor negativity, human epidermal growth factor receptors type 2 (HER2) true negativity, and a high Ki-67 labeling index of 60%. Several presumed very small daughter nodules on MRI led us to judge that her breast cancer should not be treated with breast-conserving therapy. The patient, therefore, underwent nipple-preserving mastectomy and sentinel biopsy, i.e., no metastasis on frozen section, followed by immediate breast reconstruction using an extended latissimus dorsi musculocutaneous flap. Postoperative pathological study showed that the breast cancer had similar pathological findings to those shown in preoperative pathology (Figures 1B-1D) and a higher Ki-67 labeling index of 70%. The patient recovered uneventfully and was discharged on the 9th day after the operation. The patient received dose-dense chemotherapy, i.e., epirubicin plus cyclophosphamide chemotherapy followed by paclitaxel chemotherapy, and has been well for 4 months, and is further scheduled for periodical checkups on an outpatient basis.

## Discussion

Rare coexistence of adenosis and breast cancer has been reported to date [4,5]. To the best of our knowledge, however, we could not find any reports about breast cancer metachronously developed in the histologically proven adenosis nodule. In addition, we have found that breast cancer exclusively coexisted with sclerosing adenosis but not with florid adenosis. This, therefore, is the first study that reported breast cancer metachronously developed in the florid adenosis nodule.

It remains naturally uncertain why hormone-unrelated triple negative breast cancer developed in the hormone-dependent florid adenosis nodule. The florid adenosis nodule had regressed as time passed, had shown maximum shrinkage 78 months after the VAB, and had somewhat increased in size another 24 months later, i.e., after her marriage, in this case. We speculated and misjudged that the change in endocrine environment due to her marriage was possibly related to the regrowth of the nodule. MRI evaluation at the slight nodule enlargement, if it had been done, would have enabled us to detect the breast cancer much earlier. We cannot exclude the possibility that malignant cells were present within the florid adenosis nodule at the time of VAB. However, the lesion that we followed up for a long time as a florid adenosis nodule and core needle biopsied was completely identical to the main lesion that was confirmed as triple negative breast cancer by final pathology. Therefore, marked shrinkage of the florid adenosis nodule after the VAB led us to speculate that breast cancer had newly developed within the adenosis nodule.

As mentioned above, fibrocystic disease is a disorder that has mixed epithelial and stromal component proliferation in the breast [1]. MRI and pathological findings revealed abundant fibrous components within the breast cancer in this case. It is well known that fibrous components are induced by the secretion of fibroblast growth factor (FGF) from cancer cells. FGF also activates tumor proliferation by stimulating signaling pathways such as the RAS/MAPK pathway and the PI3 kinase/AKT pathway [6,7]. The presence of fibrous components, therefore, suggested the alteration of florid adenosis nodule biology. It, however, was difficult for breast specialists to judge the presence of fibrous components in the tumor due to the absence of posterior attenuation, often observed in small masses, on ultrasound in this case.

This case has clarified that MRI is more effective than ultrasound to detect the presence of fibrous components in small masses. We, therefore, should have performed a detailed examination at 90 months after VAB when we noticed the slight tumor regrowth. It, therefore, is strongly recommended that MRI be performed when image alterations, similar to those in this case, are observed in some kind of adenosis nodules.

## Conclusions

Adenosis, including florid adenosis, is a disorder influenced by hormonal fluctuations. During the nodule shrinkage period due to the hormonal environment, ultrasound can offer good surveillance. Conversely, breast specialists should evaluate adenosis nodules with MRI when detecting slight enlargement of them to avoid under-treatment. It, therefore, is essential that breast specialists should carefully follow any type of adenosis nodules under the concept that even pathologically proven florid adenosis nodules might develop breast cancer.
